# A Muscle Physiology-Based Framework for Quantifying Training Load in Resistance Exercises †

**DOI:** 10.3390/sports13010013

**Published:** 2025-01-09

**Authors:** Frank Imbach, Stéphane Perrey, Thomas Brioche, Robin Candau

**Affiliations:** 1 Seenovate, 34000 Montpellier, France; 2 EuroMov Digital Health in Motion, University of Montpellier, IMT Mines Alès, 34000 Montpellier, France; 3 DMeM, University of Montpellier, INRAE, 34000 Montpellier, France; thomas.brioche@umontpellier.fr (T.B.); robin.candau@umontpellier.fr (R.C.)

**Keywords:** strength training, rate of force development, physiological responses, muscle fatigue, modelling, principal component analysis, force–velocity profiling

## Abstract

Background: Objective training load (TL) indexes used in resistance training lack physiological significance. This study was aimed to provide a muscle physiology-based approach for quantifying TL in resistance exercises (REs). Methods: Following individual torque–velocity profiling, fifteen participants (11 healthy males, stature: 178.36 ± 3.95 cm, and body mass (BM): 77.48 ± 7.74 kg; 4 healthy females, stature: 169.25 ± 5.03 cm, and body mass: 60.62 ± 3.91 kg) performed isokinetic leg extension exercise sessions at low, moderate, and high intensities (LI, MI, and HI, respectively). Systemic and local physiological responses were measured, and sessions were volume-equated according to the “volume-load” (VL) method. Results: Significant differences were found between sessions in terms of mechanical work (p<0.05 and p<0.001, for LI-MI and MI-HI, respectively), averaged normalised torque (p<0.001), mechanical impulse (p<0.001), and rate of force development (RFD, p<0.001 for LI-MI). RFD was mainly impacted by the accumulation of repetitions. Muscle function impairments mainly occurred at low intensities–long series, and high intensities, supported by greater RFD rate decay and changes in electromyographic activity. Therefore, accounting for muscle fatigue kinetics within objective TL indexes and using dimension reduction methods better described physiological responses to RE. Conclusions: A generic equation of muscle fatigue rise could add value to TL quantification in RE. Considering other training-related information and TL indexes stands essential, applicable to field situations and supports the multidimensional facet of physiological responses to RE.

## 1. Introduction

The rise in wearable sensors has paved the way towards athlete monitoring, a cornerstone of sports performance optimisation and injury prevention [1,2]. Based on human locomotion, these sensors mainly apply to endurance and team sports, allowing coaches and athletes to understand the exercise demand objectively [3]. However, the use of micro-technology and sensors in resistance training (RT) stands low compared to endurance and field sports. Without such support, the capture of an objective exercise demand for athlete monitoring purposes remains challenging for physical trainers and coaches [4]. Popular among amateurs and athletes aiming for fitness, performance enhancement [5], injury prevention [6] and health [7], RT induces a wide range of adaptations at the physiological [5,8], hormonal [9,10], neuromuscular [11] and cardiovascular [12] levels. In this context, one may consider a simple dose–response model in which the training dose (i.e., a quantitative representation of the mechanical work performed) induces adaptations (i.e., the response, illustrated by the adaptations mentioned above). Monitoring RT is, therefore, a prerequisite for optimising training programmes. Inappropriate training doses may indeed lead to performance impairments and injuries [13].

To date, athlete monitoring in RT relies on training load (TL) indexes. According to Wallace et al. (2009) [14] and Impellizzeri et al. (2005) [15], TL usually refers to (i) an external load defined by the mechanical work completed by the athlete, independent of their internal characteristics, and (ii) an internal load, corresponding to the psycho-physiological stresses imposed on the athlete in response to the external load. TLs are derivatives of volume and intensity parameters. The latter is quantified either in an objective way or by subjective estimates [4]. Accordingly, the method chosen to quantify TL has specific advantages and drawbacks, inherent to its nature (i.e., objective or subjective method) [4,16,17,18]. In RT, the objective quantification of volume usually refers to the total work performed within a session, while intensity relies on the average intensity of the lifting session [17]. A variety of quantification methods may be employed, including those based on relative intensities, normalisation to body mass, or the consideration of the load displacement in the calculation. From an athlete monitoring perspective, it is safe to say that internal TL indexes should reflect the body’s adaptations to exercise (i.e., external TL). Ultimately, both should be used to elucidate the athlete’s progression.

The physiological relevance of objective TL indexes in RT has been scarcely studied [19,20]. Generally, authors have found limited relevance of the simplest formulation of TL indexes (e.g., the so-called volume load, VL) [17] in terms of metabolic and hormonal responses to resistance exercise [19,20]. These results give credit to subjective methods, such as those based on ratings of perceived exertion (RPE), which correlate better with acute physiological responses [19]. However, pairwise correlations between common TL indexes (objective and subjective) remain weak or, at best, moderate [20,21]. Training load quantification methods used in RT have several limitations. First, the basic formulation of VL, which is based on the product of the number of repetitions and the intensity of the weight lifted, has a reciprocal implication. In terms of training responses, it is theoretically incorrect due to the various effects of resistance exercise intensity on physiological (e.g., fibre types I and II hypertrophic responses [8]), hormonal (e.g., growth hormone and cortisol responses [22], chronic changes in insulin-like growth factor-1, β-Endorphins, and fluid regulatory hormones changes [9]), and metabolic changes (e.g., blood lactate concentrations) [23]. Second, the movement of the load should be considered either as a weighting factor of VL or by using a mechanical work calculation to differentiate resistance exercises. Otherwise, one may encounter a rough depiction of the overall TL [16,17,20]. Third, sessional intensity is affected by the design of the training bout, such as the inter-set recovery time, which impacts training outcomes in several ways [24]. For instance, energetic metabolism benefits from more extended rest periods by recovering the adenosine triphosphate and phosphocreatine energy sources [25], while blood lactate and hormonal concentrations are also influenced [9,26]. Thus, inter-set recovery time should be considered in any TL estimates [20]. Finally, none of the above TL calculation methods (i.e., VL and derivatives, mechanical work) considers the time a muscle is held under tension (TUT) or the exercise velocity. Yet, it is known that TUT stands for a key factor of the exercise response, influencing muscle contractile properties and leading to chronic neuromuscular adaptations [27,28]. Given these limitations, the common TL quantification methods used in RT lack the requisite physiological evidence. In the context of long-term athlete monitoring, the use of approximated TL may result in practitioners failing to identify meaningful adaptations to exercise, potentially leading to flawed training prescriptions.

The objective of this study was to evaluate the accuracy of the most prevalent methods for quantifying TL, namely, the RPE, mechanical work, and their primary variants. In line with the current state of the art and by analogy with the training impulse method of Banister and Hamilton [29] applied to endurance exercises, we hypothesised that to exponentially weigh the intensity within an objective TL quantification and according to physiological observations would improve its relevance. This approach would not necessitate any specific measurement systems for athlete monitoring purposes, but it could also support their use, if any. All the training load quantification methods were evaluated in relation to a set of acute physiological responses specifically designed to assess isolated resistance exercises at sub-maximal intensity. The investigation was conducted within a controlled experimental design, with the aim of obtaining comprehensive and accurate physiological responses to exercise at the muscle level.

Following a primary exploratory analysis, two alternative approaches for quantifying relevant TL estimates for resistance exercises were proposed: (i) A TL quantification method based on individual physiological responses to exercise; (ii) A compressed representation of TL quantification methods and training-related parameters.

## 2. Materials and Methods

### 2.1. Experimental Approach to the Problem

To assess the validity of TL quantification methods regarding physiological responses under individually controlled conditions, resistance exercises were performed on an isokinetic dynamometer using concentric contractions only. The experiment was composed of a first testing session for individual-based protocol calibration and three testing sessions that involved low-, moderate-, and high-intensity resistance exercise modalities (LI, MI, and HI, respectively). These three sessions were theoretically volume-equated according to the VL method [17] and in line with previous studies [16,30,31,32,33].

### 2.2. Participants

Fifteen participants were voluntarily engaged in the study (eleven males, age: 27 ± 3.3 years, stature: 178.36 ± 3.95 cm, body mass (BM): 77.48 ± 7.74 kg, and fat mass: 11.11 ± 3.53% BM; and four healthy females, age: 21.7 ± 1.5 years, stature: 169.25 ± 5.03 cm, body mass: 60.62 ± 3.91 kg, and fat mass: 21.1 ± 5.28% BM). To be eligible, the participants had to satisfy three conditions: they had to be (i) currently engaged in resistance training with at least six months of experience prior to the start of the study, (ii) familiar with resistance exercises performed at maximal intensities, and (iii) to have no current recurrent lower limbs injury or functional limitations regarding a knee extension task performed at maximal intensity. In addition, the participants were asked to respect their usual diet all through the study period. The testing session was performed on different days but in respect of the circadian rhythm. The time between two consecutive testing sessions was fixed (>3 days) and the participants were asked to maintain their training routine without performing invasive sessions during the testing period. The study was conducted in accordance with the standards set by the declaration of Helsinki involving human subjects. Following an explanation of all the procedures, risks, and benefits associated with the experimental protocol, each participant gave his/her written informed consent prior to the experimentation. The protocol was reviewed and approved by the local research Ethics Committee (IRB-EM 2001-B, EuroMov, Montpellier, France).

### 2.3. Experimental Design

#### 2.3.1. Torque—Velocity Profile Modelling

The first testing session allowed for modelling individual torque—velocity profiles (T-V) of the quadriceps group of the dominating leg during an isokinetic leg extension task. Prior to testing, the participants completed a four-minute global cycling warm-up at 50 W and a cadence of 50 to 60 revolutions per minute on an ergocycle (Ergoselect, ergoline GmbH, Bitz, Germany).

Then, the participant was seated on an isokinetic dynamometer (Biodex system 3, Biodex Medical Systems, Shirley, NY, USA). The shaft was aligned with the axis of rotation of the knee joint to be tested. The torso, waist, pelvis and working leg were secured with straps. Handles were disposed of on either side of the chair for open-hand placement during the exercise. A shin pad attached to the distal extremity of the mechanical arm was firmly secured to the working leg about 5 cm above the medial malleolus. Once the participant was poised, lever arm amplitudes were recorded in internal to external positions (i.e., from naturally bent knee to fully extended knee, approximately zero degrees). The working leg was weighed in an external position and considered in isokinetic measurements.

A specific warm-up followed the setting step. The participants were asked to perform four repetitions of concentric extension at $1.047\;\mathrm{rad}\;s^{-1}$ with a progressive increase in intensity. Then, the participants performed two repetitions of concentric extension at maximal intensity. Since the knee extension was the only movement of interest, the knee flexion was assisted by returning to the initial position at a velocity of $5.236\;\mathrm{rad}\;s^{-1}$. After a passive rest period of four minutes, the participants performed seven series of concentric extensions, 3 min apart at the following velocities in a quasi-randomised order: $0.524\;\mathrm{rad}\;s^{-1}$, $1.047\;\mathrm{rad}\;s^{-1}$, $1.570\;\mathrm{rad}\;s^{-1}$, $2.094\;\mathrm{rad}\;s^{-1}$, $2.618\;\mathrm{rad}\;s^{-1}$, $3.142\;\mathrm{rad}\;s^{-1}$, $3.665\;\mathrm{rad}\;s^{-1}$. To limit the fatiguing effect of the lowest velocities, only two repetitions were performed at $0.524\;\mathrm{rad}\;s^{-1}$ and $1.047\;\mathrm{rad}\;s^{-1}$, against three repetitions at other velocities. These velocities were performed before the sixth of the seven series. A one-second break was set between two consecutive contractions to avoid any possible influence of the stretch-shortening cycle. The use of seven points enabled us to model a valid and reproducible T-V profile [34].

#### 2.3.2. Resistance Exercise Protocols

To assign an equated volume between the LI, MI, and HI testing sessions, the equivalent relative intensity was obtained from individual T-V profiles. The repetition maximum (RM) and their corresponding relative intensities were then estimated from a non-linear equation from Reynolds et al. (2006) [35], such as the following:

$$y=55.51\;e^{-0.0723x}+48.47.$$

Here, y denotes the percentage of relative intensity (% maximal torque) and x denotes the number of expected RM. Hence, the three sessions were performed at 58%, 77% and 93% of the theoretical maximal torque value for which the velocity is null (MVC), corresponding to 24, 9 and 3 theoretical RMs. An overview of the testing protocols is given in Supplementary Materials Table S1.

#### 2.3.3. Physiological Responses and Data Collection

In order to match recordings on a single time frame, mechanical (position, velocity, and torque), cardiovascular, and neuromuscular measurement systems were coupled using analogue signals (Trigano Analog Input Adapter, Delsys, Natick, MA, USA).

### 2.4. Systemic Measurements

#### 2.4.1. Cardiac Measurements

The participants wore two ECG sensors (Trigno EKG Biofeedback, Delsys, MA, USA) for a continuous measure of heart rate (HR) activity. Prior to starting the experiment, the quality of the HR activity recording was visually checked over the Q-, R- and S-waves displayed in real time on the EMGworks software (version 4.8.0, Delsys, MA, USA). Heart rate was further extracted from the R-R intervals. The continuous signal was then averaged using a 10 s bin moving average filter. The rate decay of HR during recovery was estimated using a mono-exponential function

(1) $$f\left(x\right)=be^{-\alpha x}+c\;,$$

with b being a gain constant, c denotes an intercept, and α a negative constant for exponential decay.

#### 2.4.2. Pulmonary Gas Exchange Measurements

Breath-by-breath gas exchanges were analysed through a portable metabolic cart (k4b^2^, Cosmed, Rome, Italy), previously validated by several independent authors in locomotor activities [36]. Before each session, the portable system was powered on to warm up for 10 min. Calibration of the oxygen ($O_{2}$) and carbon dioxide (${\mathrm{CO}}_{2}$) analysers was performed before every test using two-point calibration with two precision-analysed gas mixtures (room air and a high-precision certified calibration tank gas containing O_2_ 16%, CO_2_ 5%, and balance nitrogen). Turbine flow calibration was determined using a high-precision 3 L calibration syringe in an eight-pump series. For the subsequent numerical analysis, the recorded breath-by-breath gas exchange measurements were linearly interpolated on a second-by-second basis. A moving average filter was applied to the raw data to obtain an exploitable signal. From the net pulmonary oxygen uptake ($\dot{{\mathrm{VO}}_{2}}$) and considering the major contribution of glycolytic pathways during exercise, we estimated the net energy expenditure (EE) according to an energy equivalent of 21.3 J per millilitre of $O_{2}$ [37].

During exercise, the rate of $\dot{V}O_{2}$ was computed from the linear relationship between $\dot{V}O_{2}$ and time. At rest, the rate of $\dot{V}O_{2}$ recovery was given by the generic mono-exponential function defined in Equation (1).

#### 2.4.3. Metabolic and Hormonal Measurements

Blood lactate concentrations $\left[lact_{b}\right]$ in $\mathrm{mmol}\cdot L^{-1}$ were collected four times during each testing session using a finger prick and a valid hand-held lactate analyser (Lactate Pro, KDK Corporation, Arkray, Kyoto, Japan) [38]. The first sample was collected after the participant was fully equipped and prior to any exercise. A second sample was taken at the onset of the testing (both global and specific warm-ups being completed). Changes in $\left[lact_{b}\right]$ were evaluated at 1 min and 3 min post-exercise to cover several possible kinetics of $\left[lact_{b}\right]$ responses following the exercise.

In addition, 100 µL to 300 µL of blood was taken at the fingertips using a lithium heparin 500 LH Microvette (Sarstedt, Nümbrecht, Germany) for plasma cortisol concentration ($\left[cort_{p}\right]$) analysis. Immediately after collection, the samples were centrifuged for 10 min at 2000 $\mathrm{rev}\cdot \min^{-1}$. Then, plasma (50 µL to 150 µL) was collected from the centrifuged sample and stored at −80 °C. Plasma cortisol analysis was performed twice (10 µL) using enzyme-linked immunosorbent assay kits (Cortisol ELISA, Minneapolis, MN, USA). 

### 2.5. Local Measurements

#### 2.5.1. Mechanical Measurements

For any exercises, torque (Nm), angular velocity ($rad\;s^{-1}$), and position (rad) were recorded at a 148 Hz sampling frequency. From the torque production over time, we extracted the rate of force development (RFD) values from the onset of exercise to 100 ms, peak RFD, and mechanical impulse over the entire repetition (${\mathrm{RFD}}_{0–100}$ and ${\mathrm{RFD}}_{\mathrm{peak}}$ in $Nm\cdot s^{-1}$, IMP in Nm·s, respectively).

#### 2.5.2. Skeletal Muscle Oxygenation and Oxidative Function Measurements

Locally, the skeletal muscle oxidative capacity of the vastus lateralis (VLat) was evaluated by in vivo near-infrared spectroscopy (NIRS). The use of NIRS, which has gained popularity in sports applications since the early 2000s [39], is considered a valid method for evaluating skeletal muscle oxygenation and oxidative metabolism [40,41]. The portable NIRS device (PortaLite, Artinis Medical Systems BV, Einsteinweg, The Netherlands) used in this study was a continuous dual-wavelength system that simultaneously uses the modified Beer–Lambert and spatially resolved spectroscopy (SRS) methods. Changes in myoglobin were assumed to be small compared to haemoglobin [42]. Changes in tissue oxyhaemoglobin, deoxyhaemoglobin, and total haemoglobin concentration ($\Delta \left[O_{2\mathrm{Hb}}\right]$, $\Delta \left[\mathrm{HHb}\right]$, and $\Delta \left[\mathrm{tHb}\right]$, respectively) were measured using the difference in absorption characteristics of light at 750 and 850 nm. The tissue saturation index (TSI) was calculated using the SRS method. Skinfold measurement at the NIRS optodes location was carried out prior to the first session to ensure valid measurements regarding the adipose tissue thickness. This allowed us to determine an oxygenation index ($\Delta \left[{\mathrm{Hb}}_{\mathrm{diff}}\right]$) for the subsequent analysis.

From the $\Delta \left[{\mathrm{Hb}}_{\mathrm{diff}}\right]$ measurements, we estimated the muscle oxygen consumption $m\dot{V}O_{2}$ through the rate decay of $\Delta \left[{\mathrm{Hb}}_{\mathrm{diff}}\right]$ during the most representative of the first repetitions per series, in which the ischemia arterial occlusion remains unchanged [43].

#### 2.5.3. Neuromuscular Measurements

The activity of the VLat, vastus medialis (VMed), and rectus femoris (RFem) were assessed through surface electromyography (EMG) using three sensors (Trigno Avanti, Delsys, MA, USA) located in respect of the SENIAM recommendations [44]. Electrode sites were properly shaved and cleaned with alcohol before electrode placement. The sampling frequency of the EMG signals was set at 2048 Hz, recorded through the EMGworks software (version 4.8.0), and exported using the Delsys file utility application (Delsys, MA, USA). The activity of quadriceps muscles was analysed in both time and frequency domains. In the time-domain analysis, the integrated signals amplitude was calculated from VLat, VMed, and RFem for each knee extension using a root mean square (RMS) function (see Equation (2)), following a signal rectification and filtering using a second-order low-pass Butterworth filter with a cut-off frequency of 10 Hz. Then, normalisation to the mean signal computed from the first repetition and a time-normalisation were processed, ensuring unbiased within-session and within-participant analysis [45].

(2) $$f_{RMS}=\underset{T\to \infty }{\lim}\sqrt{\frac{1}{2T}\;\int_{-T}^{T}{\left[f\left(t\right)\right]}^{2}dt.\;}$$

In frequency-domain analysis, and because the testing exercises involved dynamic contractions, short-term Fourier transform (STFT) was processed on 125 ms overlapping samples of length l=250 ms. Then, a power spectral density (PSD) representation allowed for the extraction of median frequencies (MDFs) to detect impairment in EMG signals due to muscle fatigue [46]. It is defined in the following:

$$\sum_{j=1}^{MDF}P_{j}=\sum_{j=MDF}^{M}P_{j}=\frac{1}{2}\sum_{j=1}^{M}P_{j}$$

where $P_{j}$ is the EMG power spectrum at a frequency bin j, and M is the length of the frequency bin [46].

**Figure 1:**
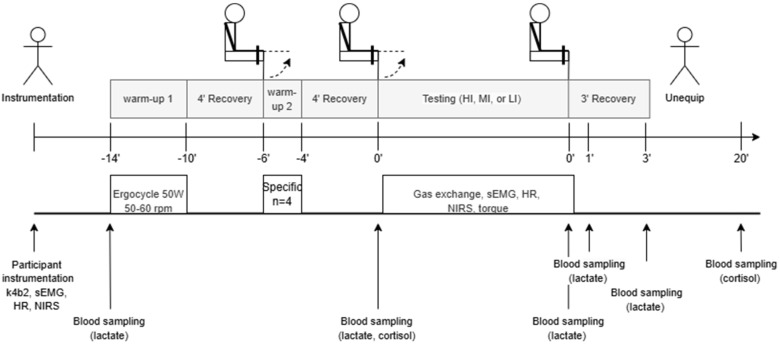
Diagram of the experimental workflow. Hi, MI, and LI denote the three resistance testing sessions. The participants performed passive recovery sequences within the sessions. Dotted arrows indicate concentric knee extension.

To summarise, the experimental workflow is illustrated in Figure 1.

### 2.6. Statistical Analysis

First, normality and variance homogeneity of the residual errors were checked by a Shapiro–Wilk and a Levene tests, respectively. The distributions of the physiological responses across the three testing sessions were then compared through ANOVAs followed by Tukey’s post hoc analysis. The marginal mean differences $\beta_{diff}$ were reported for comparisons. Effect size from ANOVAs was reported as $\eta^{2}$ within 95% confidence intervals (CIs).

Linear mixed models (LMMs) were computed to assess the contribution of the variables related to each resistance exercise protocol, with training-related parameters as fixed effects, and participants as a random effect. Due to the small sample size and weak statistical power for a desired effect size and significance level (P ≈ 20% from a post hoc analysis, considering a moderate effect size such that ${Cohen}^{\prime }s\;d=0.5$, n=15, and k=3), we conducted the analysis in a Bayesian framework. A priori information over parameter distribution was provided based on empirical knowledge and the literature. The Hamiltonian Monte Carlo algorithm was used to infer the model parameters. Particular attention has been given to model diagnosis and convergence of Monte Carlo Markov Chains (MCMC) [47]. Formally, the model is defined as

$$Y_{ij}=X_{ij}\beta +Z_{ij}b_{j}+\epsilon_{ij},$$

where $Y_{ij}$ denotes the outcome for individual i in group j, $X_{ij}$ denotes the row vector of fixed-effect predictors for individual i in group j, is the column vector of population-level coefficients, $Z_{ij}$ denotes the design matrix for group-specific (random) effects, $b_{j}$ denotes the column vector of group-specific effects, and $\epsilon_{ij}$ is the residual error. Based on empirical assumptions and the literature, univariate Gaussian and weakly informative priors are specified on population-level effects, such that $\beta ∼N\left(0,10\right)$; while weakly informative priors on standard deviations and correlations of the group-specific effects following a Student-t distribution, $b_{0j}∼\text{Student-t}\left(1,0,2.5\right)$ and $b_{1j}∼\text{Student-t}\left(1,0,5\right)$ for intercept and slope, respectively. For the four Markov chains, we considered 4000 iterations, a warm-up of 1000 iterations, and thin = 1. Note that for the RFD and time-domain electromyographic analysis, less restrictive priors have been considered due to greater expected estimates on posterior distributions. Hence, we used $\beta ∼N\left({0,k10}^{2}\right)$ with *k* = 2 for population-level and group-specific effects. The Bayesian models have been written in *Stan* and using the *brms* R package [48].

Quantitative variables are standardised for modelling and the model estimates (β) are reported within 95% credible intervals (CIs).

Lastly, a principal component analysis (PCA) was performed to build a linear combination of the initial variables that maximises the variance onto orthogonal axes. A compressed representation of the data was either determined by the first principal component (PC), or a combination of the most contributing PCs using a meta-regression model.

## 3. Results

In this section, we sequentially present the physiological responses to exercise accounting for resistance exercise parameters and individual T-V profiles.

### 3.1. Neuromuscular Responses

#### 3.1.1. Neuromechanics

**Figure 2:**
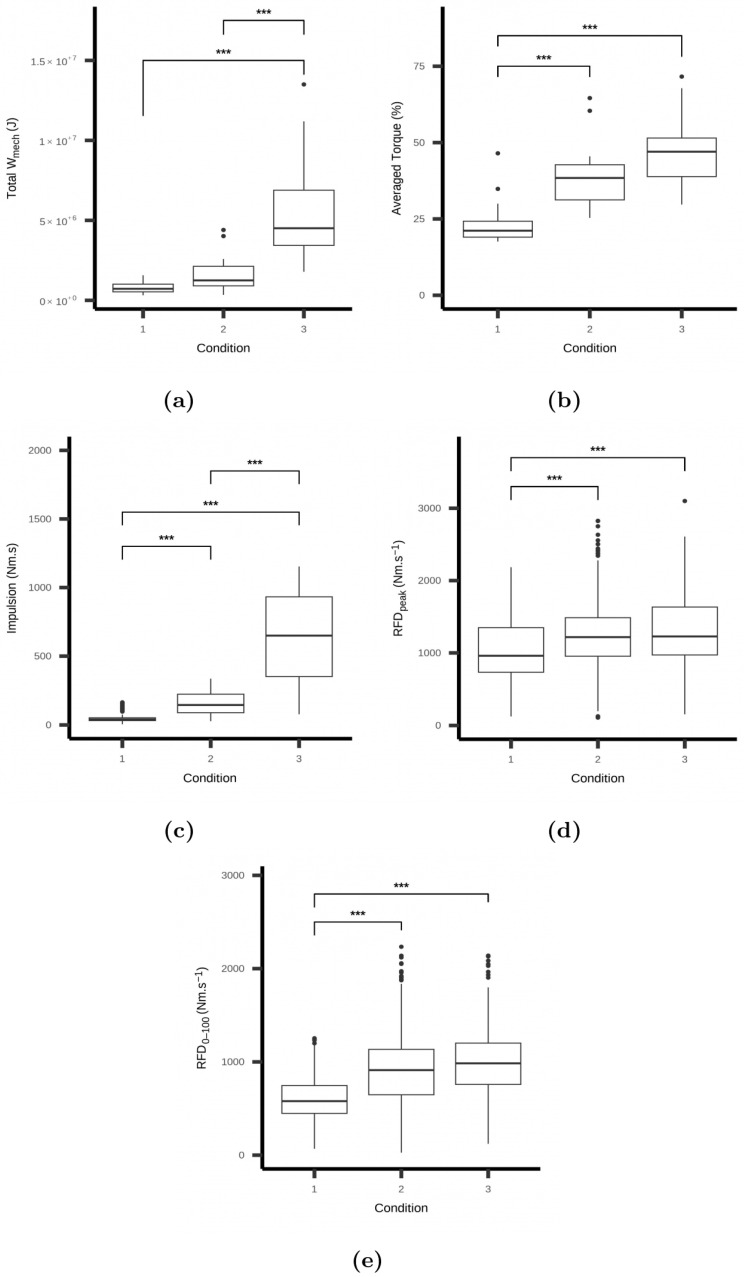
Distributions of the total mechanical work ($W_{\mathrm{mech}}$), normalised averaged torque, impulsion, ${\mathrm{RFD}}_{\mathrm{peak}}$, and ${\mathrm{RFD}}_{0–100}$ across the testing sessions. Plain circles represent extreme values, and the horizontal box line shows the median of the distribution. Asterisks denote the significance level (*** stand for *p* < 0.001). Note that conditions 1, 2, and 3 refer to low-, moderate-, and high-intensity resistance exercise sessions.

A first analysis of the mechanical measurement distributions showed significant differences in terms of mechanical work, normalised averaged torque, and mechanical impulse between the three testing sessions (see subfigures in Figure 2). As expected, exercises performed at higher relative intensities—associated with a lower exercise velocity and hence, a greater TUT—induced the greatest values. Moderate to strong positive correlations were, thus, found between the total mechanical work on the one hand and averaged torque and mechanical impulse on the other ($r=0.594\in \left[0.364,0.756\right]\;95\%\;CI,p=0.001$ and $r=0.762\in \left[0.604,0.863\right]\;95\%\;CI,p<0.001$ for the averaged torque and mechanical impulse, respectively).

An intra-session analysis showed that the torque produced likely decreased with the accumulation of repetitions ($\beta =-1.85\in \left[-2.91,-0.74\right]\;95\%\;CI$). Yet, this is not consistent across the testing sessions. An interaction between the protocol and the accumulation of repetitions over the individual torque response suggests that higher relative intensities (i.e., MI and HI) may induce positive changes. Details are provided in Table S2.

An overview of the averaged RFD over the testing sessions indicated that ${\mathrm{RFD}}_{\mathrm{peak}}$ and ${\mathrm{RFD}}_{0–100}$ significantly increased between the LI and MI sessions (*p* < 0.001). However, changes remain not significant between the MI and HI sessions despite a large increase in exercise intensity (see subfigures in Figure 2). The within-session analysis showed that performing numerous repetitions within or across series lowered both ${\mathrm{RFD}}_{\mathrm{peak}}$ and ${\mathrm{RFD}}_{0–100}$ ($\beta =-68.50\in \left[-120.16,-15.30\right]\;95\%\;CI$ and $\beta =-66.49\in \left[-108.96,-24.27\right]\;95\%\;CI$, respectively).

In addition, an interaction between the testing session (i.e., the relative intensity) and the accumulation of repetitions showed that MI had possibly a greater sustained RFD than HI and LI.

Having a decreasing effect of the repetitions’ accumulation over ${\mathrm{RFD}}_{\mathrm{peak}}$ and ${\mathrm{RFD}}_{0–100}$ suggests that exercise induces the progressive impairment of neuromuscular function. However, changes in IMP did not evoke any significant decay across repetitions. For the three outcome variables, the models attributed a significant portion of the variance to random effects, as indicated by PVE, ICC, and $R^{2}$, while achieving strong overall explanatory power. However, the PVE on slopes contributes only slightly to explaining the variance, and a random intercept-only structure could capture the inter-subject differences and achieve similar performance ($BF_{10}\in [0,1]$). Details about the model estimates and summary are given in Table 1.

On this basis, it is possible to estimate the rate of muscle fatigue occurrence from the individual regression slopes. The low-intensity condition LI showed a homogeneous distribution of the RFD rate decays, suggesting a relatively consistent apparition of muscle fatigue across participants.

In contrast, the MI and HI sessions showed a greater variability in the ${\mathrm{RFD}}_{\mathrm{peak}}$ and ${\mathrm{RFD}}_{0–100}$ rate decays across participants, supporting the singularity in response to exercise at the theoretical MI and HI modalities. We note that, according to the average population studied, the distributions of the RFD slopes are not significantly different between LI and MI (p>0.05, see Figure S1). 

#### 3.1.2. Electromyographic Activity

In the time domain, an amplitude of EMG signals from knee extensors was given by the linear combination between the RMS values computed from the averaged VLat, VMed, and RFem signals. Assuming that resistance exercises might induce muscle fatigue, we first investigated the contribution of performing multiple sets (in the MI and HI sessions) to a potential muscular fatigue apparition.

Changes in the EMG amplitude distributions over repetitions were in favour of a small increase in the RMS values over repetitions ($\beta =12.56\in \left[2.45,23.01\right]\;95\%\;CI$, see Table S3) and a slight decrease in normalised averaged torque, as expected and seen in Subsection “3.1.1. Neuromechanics” ($\beta =-1.85\in \left[-2.91,-0.74\right]\;95\%\;CI$).

Considering only testing conditions with multiple series (MI and HI sessions), we found that the average RMS computed across sets mostly decreased during MI ($\beta =-40.78\in \left[-67.57,-13.08\right]\;95\%\;CI$). As with exercise intensity, the effect of exercise velocity was likely positive on changes in leg extensor RMS rate decays ($\beta =0.41\in \left[0.06,0.76\right]\;95\%\;CI$). However, the heaviest session, HI, suggested a positive effect on the RMS slopes ($\beta =38.85\in \left[4.15,71.96\right]\;95\%\;CI$). No interaction effect between the testing setup and the exercise velocity appeared ($\beta =-0.29\in \left[-1.38,0.82\right]\;95\%\;CI$). Hence, the results indicate that performing slower repetitions impaired negatively the RMS rate decays for exercises performed at moderate intensity.

In the frequency domain, we observed a slight downward shift in average the MDF values over VMed, VLat, and RFem muscles with the accumulation of repetitions ($\beta =-0.45\in \left[-0.61,-0.29\right]\;95\%\;CI$). Such a decrease was likely dependent on testing conditions, with little decreasing effect of repetitions at high intensities (i.e., HIs) and a positive effect at moderate intensities (i.e., MIs). Again, group-specific effects through random intercepts and slopes bring significant information on the model variance ($BF_{10}=10.80$, $ICC=0.53\in \left[0.34,0.73\right]95\%\;CI$). See Table 2 for details.

From the STFT samples, the slopes of MDF averaged over muscles ($\bar{\mathrm{MDF}}$) showed a greater magnitude of muscle function impairments at high intensities (see Figure S2) which is likely supported by the mixed effect regression ($\beta =-2.37\in \left[-3.08,-1.65\right]\;95\%\;CI$, see Table 2)

### 3.2. Metabolic and Hormonal Responses

#### 3.2.1. Blood Lactate Concentrations

Metabolic responses to exercise showed that changes in $\left[lact_{b}\right]$ were mostly influenced by the individually fitted protocol. The session performed at low intensity and associated with a single set—high volume—induced the greatest changes in $\left[lact_{b}\right]$ regarding the baseline values. On the other hand, the higher the exercise intensity, the lower the variation in $\left[lact_{b}\right]$ after exercise completion ($\beta =-8.30\in \left[-13.07,-3.13\right]\;95\%\;CI$ compared to LI session). In addition, an interaction between the testing condition and the exercise velocity indicated smaller changes in $\left[lact_{b}\right]$ in response to high-intensity—low velocity—exercises. Full details are provided in Table S4.

#### 3.2.2. Plasma Cortisol Concentrations

The distributions of $\left[cort_{p}\right]$ did not show any significant differences between the three testing conditions. Considering the experimental design, neither the LI, nor the MI and HI sessions significantly induced a noticeable hormonal stress state when $\left[cort_{p}\right]$ was measured at five minutes post-exercise. Details are provided in Table S5.

### 3.3. Cardiac and Pulmonary Gas Exchange Kinetics

#### 3.3.1. Heart Rate

One-way repeated measures ANOVAs indicated that the distributions of the post-exercise HR slopes computed from Equation (1) were significantly lower within HI than LI ($\beta_{diff}=-0.018\in \left[-0.033,-0.003\right]\;95\%\;CI,p<0.05\;,\eta^{2}=0.08\in \left[0.01,0.18\right]\;95\%\;CI$). In addition, the distributions of recovery amplitudes were significantly different only between HI and MI ($\beta_{diff}=-10.199\in \left[-19.112,-1.286\right]\;95\%\;CI,p<0.05\;,\eta^{2}=0.09\in \left[0.01,0.18\right]\;95\%\;CI$).

#### 3.3.2. Oxygen Uptake Measurements

At exercise, the distributions of the average rate of $\dot{V}O_{2}$ were not significantly different between the testing conditions (all the coefficients close to 0 along with negative to positive 95% credible intervals), despite substantial differences in terms of exercise intensity and repetitions.

**Figure 3:**
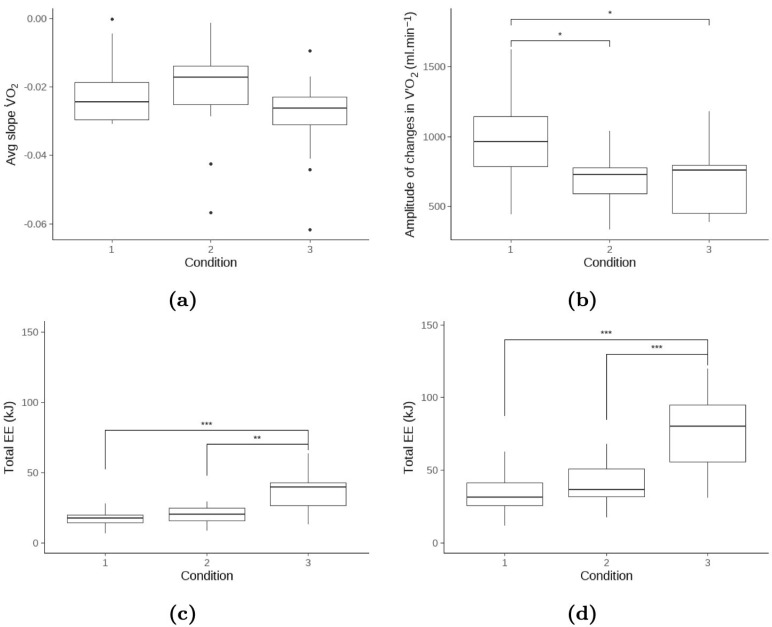
Distribution of (**a**) session-averaged $\dot{V}O_{2}$ slopes and (**b**) session-averaged $\dot{V}O_{2}$ amplitudes computed over recovery phases; (**c**) total energy expenditure from exercise phases only; and (**b**) total energy expenditure from exercise and post-exercise recovery phases. Plain circles represent extreme values, and the horizontal box line shows the median of the distribution. Asterisks denote the significance level (***, **, and * stand for *p* < 0.001, *p* < 0.01 and *p* < 0.05, respectively). Note that conditions 1, 2, and 3 refer to low-, moderate-, and high-intensity resistance exercise sessions.

A similar observation was made post-exercise, where the $\dot{V}O_{2}$ slopes averaged over the session were not significantly different between the testing conditions. However, the amplitudes of $\dot{V}O_{2}$ averaged over each session showed greater amplitudes at recovery for LI, which were associated with higher $\dot{V}O_{2}$ values at the onset of the recovery phase ($p=0.018,\;\eta^{2}=0.23\in \left[0.03,0.41\right]\;95\%\;CI$, see Figure 3).

In analogy with the total mechanical work, we observed a significant increase in total energy expenditure from the $\dot{V}O_{2}$ measurements across the testing conditions (see subfigures in Figure 3). Naturally, such metabolic measurements are mostly impacted by the magnitude of $\dot{V}O_{2}$ as an index of exercise intensity and the exercise duration ruled by the total number of repetitions and TUT.

### 3.4. Muscle Tissue Oxygenation

Locally and during exercise, the distributions of the $\Delta \left[{\mathrm{Hb}}_{\mathrm{diff}}\right]$ rate decays showed greater shifts within the LI and MI testing protocols (see Figure S3). Changes in TSI showed a similar pattern. However, neither the testing condition, nor the exercise velocity seemed to impact the amplitudes of $\Delta \left[{\mathrm{Hb}}_{\mathrm{diff}}\right]$ and changes in the TSI values.

### 3.5. Relationships Between Training Load Indexes and Physiological Responses

#### 3.5.1. Using an Estimation of Muscle Fatigue as a Weighting Factor of Objective Training Load Indexes

In analogy with the training impulses of Banister and Hamilton [29], we defined a new model based on neuromuscular impairments measured at different exercise intensities of resistance exercise. In this context, RFD appears to be (i) a relevant indicator of fatigue apparition and neuromechanics impairments according to the results presented in Subsection “3.1. Neuromuscular responses” and supported by the literature [11,49], and (ii) a practical, raising, and non-invasive parameter that benefits from the recent technological improvements in measurement systems (e.g., linear position transducers and inertial measurement units).

**Figure 4:**
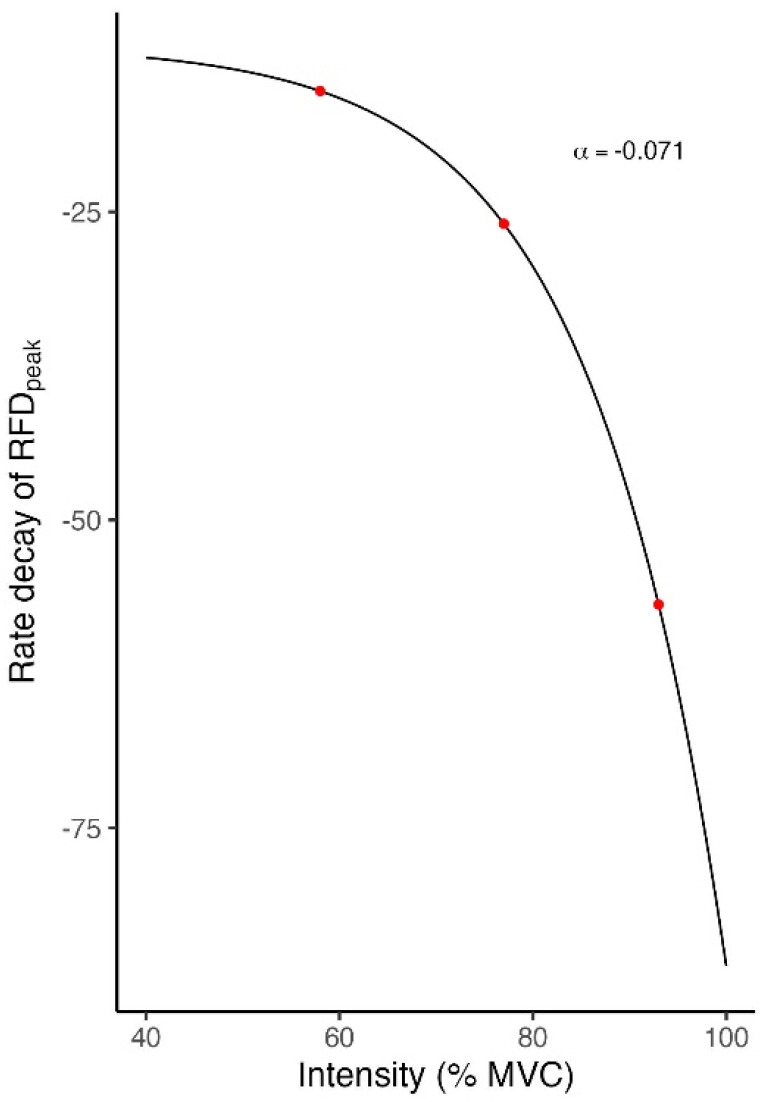
Representation of the non-linear relationship between the rate decay of ${\mathrm{RFD}}_{\mathrm{peak}}$ and the relative exercise intensity (red dots at 58 %, 77 %, 93 % Maximal Voluntary Contraction, MVC).

From the averaged rate decays of ${\mathrm{RFD}}_{\mathrm{peak}}$ observed during exercise, we modelled the non-linear relationship between ${\mathrm{RFD}}_{\mathrm{peak}}$ and exercise intensity (see Figure 4) according to a mono-exponential function (see Equation (1))

This relationship allowed for considering a neuromuscular function that is exponentially impaired by exercise intensity. Hence, we defined three formulations of a ${\mathrm{RFD}}_{\mathrm{peak}}$-based model of TL quantification in the following:

(3) $$TL_{RFD}=V\;I\left(\frac{1}{e^{\alpha \;I}}\right)$$

$$TL_{RFD}=V\;Ie^{-\alpha I},$$

where V is the number of repetitions performed,  I denotes the relative intensity (% MVC), and α the rate decay such as α=−0.071. From Equation (3), we write its density correspondence

(4) $$TL_{RFD_{d}}=\frac{TL_{RFD}}{R}\;,$$

with *R* being the total inter-set recovery duration.

Finally, and like RFD, the IMP measure is becoming increasingly accessible. It could be used as a surrogate for the product of volume and number of repetitions. Hence, $TL_{RFD}$ becomes

(5) $$TL_{RFD^{*}}=\sum_{n=1}^{N}\int_{s=1}^{S}T\;ds\;e^{-\alpha \;I}\;,$$

with N being the number of repetitions, *S* denotes the duration of each repetition, and *T* is the torque produced.

#### 3.5.2. A Linear Combination of Quantification Methods and Exercise Related Variables

From the results presented so far, we have processed a PCA based on training-related features, the usual TL indexes and the three ${\mathrm{RFD}}_{\mathrm{peak}}$-based models presented in Subsection “Using an estimation of muscle fatigue as a weighting factor of objective training load indexes”.

**Figure 5:**
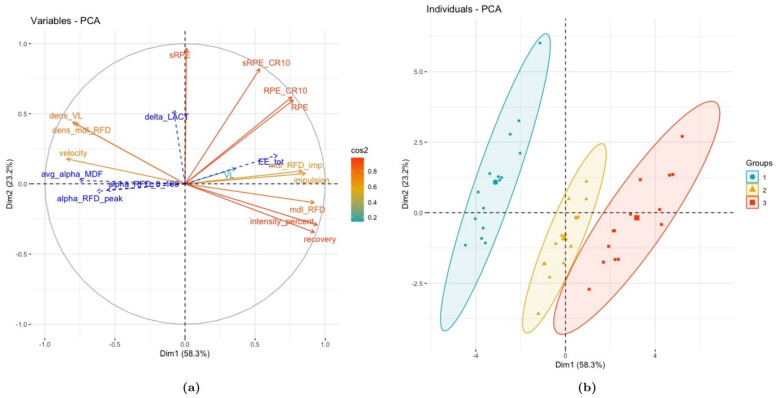
Principal component analysis with (**a**) the circle of correlation and (**b**) the projection of individuals. In (**a**), the variables in blue are illustrative and not accounted for in the calculation of the distance between individuals. In (**b**), clusters 1, 2, and 3 over the testing sessions represent the LI, MI, and HI sessions, respectively.

The first two dimensions express 81.5% of the total data set inertia (58.3% and 23.2% explained by the first and second dimensions, respectively). Graphically, the circle of correlation in Figure 5 shows the correlated features along the first and second axes.

Of the physiological responses observed above, only changes in $\left[lact_{b}\right]$ were likely to be represented in the second dimension. In contrast, MDF, RFD-related ones, and EE were mainly represented in the first dimension.

Hence, the RPE-related variables seemed to be the key indicators of $\left[lact_{b}\right]$ responses, whereas the other variables were likely better suitable to explain neuromuscular and cardio-respiratory responses. In addition, the TL methods represented through their density (i.e., dens_VL and ${\mathrm{dens}\_\mathrm{TL}}_{\mathrm{RFD}}$) were likely correlated and anti-correlated with other objective TL indexes and training-related parameters (see Figure 5).

From the projection of the individuals, we identified each cluster that maps with the three testing protocols (see Figure 5). Each of the clusters was well represented on the first dimension, while the second dimension likely depicted the dispersion of the individuals’ projection for each protocol.

**Figure 6:**
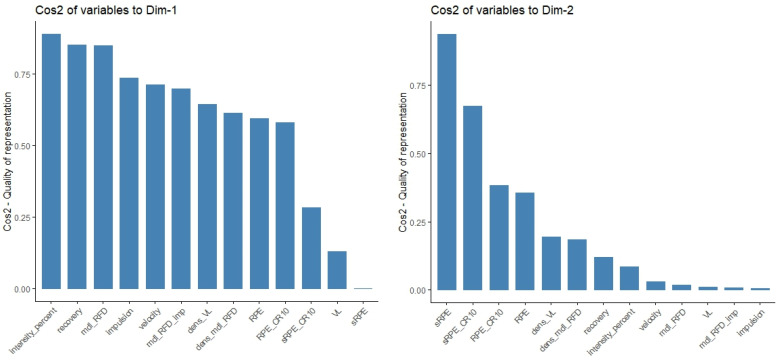
Contributions of training-related parameters and training load quantification methods in the first two principal components.

Similarly to the physiological responses, Figure 5 showed that the RPE-related indexes mostly contributed to explaining the individuals projected on the second dimension. On the contrary, the other variables were more likely to represent individuals projected on the first dimension and explained a more significant part of the total variance. The contributions of the features within the dimension are displayed in Figure 6.

#### 3.5.3. Relationship Between Training Load Quantification Methods and Physiological Responses

Linear relationships between each TL quantification method and the main physiological responses to resistance exercise are provided in Table 3. Globally, compressing information from different training load quantification methods into the first two PC explained the greatest part of the variance in the physiological responses through linear relationships. One notch below, the RPE-based methods provided moderate $R^{2}$ scores, but still bring more information than the VL methods.

## 4. Discussion

The main objective of this study was to evaluate the accuracy of common TL quantification methods regarding a set of physiological responses to resistance exercises, and to provide evidence-based alternatives. Based on a first exploratory analysis, we will first discuss the physiological responses observed during and after resistance exercises. Then, we will review the relevance of all the TL quantification methods investigated in the study to the key physiological responses.

### 4.1. Physiological Responses to Various Resistance Exercise Protocols

First, differences in terms of mechanical measurement across the testing sessions (i.e., averaged torque, total $W_{\mathrm{mech}}$, RFD and impulse, RFD, etc.) were expected, since higher exercise intensity results in a greater force production corresponding to higher levels of muscle activation [50].

In addition, we observed a decrease in the torque produced during exercise in the low-intensity test (i.e., LI), suggesting an accumulation of fatigue through repetitions. While this was not noticeable for higher intensities, a reasonable explanation might come from a longer recovery time between sets and shorter series performed at moderate to high intensity.

Similarly, the protocol design may partly explain the heterogeneity in RFD responses to exercise observed across the testing sessions. Factors such as relative intensity, interset recovery time, and total number of repetitions could influence the sustainability of RFD throughout the exercise series. Additionally, the variability in neuromuscular impairment among the participants might be attributed to the heterogeneity of the population, which includes individuals with diverse training backgrounds [51]. Such results highlight that the neuromuscular responses are singular, protocol design dependent, and therefore, multifactorial [52,53,54].

The downward shift of ${\mathrm{RFD}}_{\mathrm{peak}}$ and ${\mathrm{RFD}}_{0–100}$ over the accumulation of repetitions suggests the impairment of the neuromuscular function with an increase in exercise repetitions. This is in line with the literature, as force generation (including the rate of RFD) and inorganic phosphate release are closely related [55]. Indeed, under muscle fatigue, ions H+ and inorganic phosphate concentrations increase in the myoplasm, impairing the strong bindings in the actomyosin complex and inhibiting the release of calcium in the sarcoplasmic reticulum (i.e., the excitation–contraction coupling). These chemo-mechanical changes, therefore, result in a decrease in force production [56,57]. On this basis, RFD has been considered a key indicator of neuromuscular fatigue [49,54]. Furthermore, it offers an alternative to repetition maximum-based prescriptions given that the impairments of movement are anticipated under fatigue, manifested as a reduction in force production and, consequently, velocity and/or motor control. Practitioners can, therefore, modify the demand in accordance with specific and tailored exercise velocities. Regarding mechanical impulse, our findings indicate that it does not reflect neuromuscular responses such as the muscular fatigue observed in the early phase of RFD. However, it is indicative of mechanical work production.

A concurrent decrease in torque output and increase in averaged EMG signals over repetitions indicates neuromuscular adaptations to strenuous exercise [58]. Among the potential causes of such neuromuscular changes, some authors have reported a high correlation between the decline of peak torque and the percentage of type II fibres [59,60] and an increase in muscle lactate [60].

We note that adaptation mainly concerns the first testing session (i.e., LI) based on a single set of twenty-four repetitions performed at an intensity close to 60% of the theoretical MVC. The results support the fact that the relationship between EMG amplitude according to RMS and the torque produced is non-linear (or at least quadratic) [61,62,63]. This might be related to (i) the fusion of individual motor units (MUs), and the subsequent tetanus phenomena that occur between 60% and 80% of MVC [62], and (ii) the fact that the number and amplitude of recruited MUs are not directly related to changes in isokinetic exercise velocities [61]. In addition, the large and credible effect of exercise velocity over EMG-RMS responses is in agreement with the literature [61].

Carried out at the highest relative intensity, performing shorter sets of repetitions with longer interset recovery time (i.e., HI condition) tends to inhibit such neuromuscular adaptations. This was expected since four minutes of passive rest between three theoretical RM exercises would allow a substantial recovery of neuromuscular function [26]. However, the EMG analysis in the frequency domain underlines that HI sessions induced the most significant magnitude of muscle function alteration. Specifically, the mechanisms behind the decline of MDF can be attributed to (i) a fall in conduction velocity throughout repetitions and (ii) the muscle phenotype and particularly its fibre type distribution [64]. Furthermore, such neuromuscular adaptation could be an essential factor in exercise realisation, optimising force and ensuring the economical activation of fatigued muscle by the central nervous system [65].

Regarding the metabolic responses to exercise, changes in $\left[lact_{b}\right]$ also agree with the literature since the lactate response is a function of exercise intensity, volume (according to the accumulation of exercise repetitions), TUT, and inter-set recovery time [23,66]. As expected, exercises performed at moderate to high intensities, with a moderate to large number of repetitions and short recovery time, induced the greatest changes in $\left[lact_{b}\right]$ [20].

With respect to $\left[cort_{p}\right]$, none of the three testing conditions elicited significant modifications. Our results do not corroborate previous findings in which $\left[cort_{p}\right]$ was substantially impacted by resistance exercise performed at moderate intensity and associated with a high volume and low resting periods [19], even in isokinetic conditions [67]. In our study, the participants performed only a small number of repetitions (equal to or less than 24 repetitions) in a concentric mode only. Therefore, we can safely suppose that our protocols were not strenuous enough to elicit noticeable hormonal stress as measured by $\left[cort_{p}\right]$.

The cardio-pulmonary results indicate that HR kinetics slightly differ between the three testing sessions. Shorter time courses of HR kinetics are observed at high exercise intensities, whether HR is measured during exercise or recovery. However, the magnitude of HR differences between the protocols remains marginal and points out the negligible impact of localised resistance exercise on cardiac function.

In terms of oxygen uptake, the slight elevation of $\dot{V}O_{2}$ reported in our study is consistent with the literature [53] and highlights the weak contribution of an isolated muscle group on cardio-pulmonary function [68]. Assuming that higher-intensity sessions do not induce substantial changes in $\dot{V}O_{2}$, our results also corroborate the changes in $\left[lact_{b}\right]$ (a proxy of the anaerobic glycolysis contribution to energy supply), for which the changes were significantly greater for LI and MI than HI sessions. This suggests that performing fewer repetitions at higher intensities does not induce an elevation of $\dot{V}O_{2}$ during exercise and, therefore, no significant changes in anaerobic metabolism contribution to task completion, which also supports previous findings [69]. However, $\dot{V}O_{2}$ observed should be interpreted with caution since breath irregularities and apnea times occurred during exercises performed at low velocities.

Locally, a proxy of $\dot{V}O_{2}$ was estimated through the rate of $\Delta \left[{\mathrm{Hb}}_{\mathrm{diff}}\right]$, which is a more suitable measurement of oxygen consumption at the level of a muscle group [43,70] than $\dot{V}O_{2}$. A correlation is naturally expected between the local and systemic measurements. However, apnoea times that occurred at the lowest exercise velocities impaired $\dot{V}O_{2}$ measurements and their relationship with $m\dot{V}O_{2}$.

### 4.2. Training Load Indexes and Their Relationship with Physiological Responses

From a new space of dimensions, PCA reveals that (i) objective TL indexes and mechanical training-related parameters (e.g., exercise velocity and intensity, recovery time, impulse) can represent an average response for a given testing condition, and (ii) subjective TL indexes (i.e., RPE-based features) are likely to discriminate an inter-individual dispersion (see Figure 5).

This is in line with the literature since the subjective measures of TL provide extra individual information as a psychophysiological integrator [71], able to differentiate training responses between individuals for a given external TL.

Upon a closer examination of the explanatory power of the TL methods with respect to a set of key physiological responses to resistance exercise (see Table 3), it becomes evident that the common VL method [17] is severely lacking in its ability to describe the physiological responses. That was indeed expected, since the testing sessions were theoretically volume-equated using the VL method, whereas the participants showed heterogeneous T-V profiles and different exercise velocities. This questions the physiological relevance of the VL index for training programming purposes. However, accounting for interset recovery time through a weighted representation of VL (dens_VL) along with other TL parameters likely improved its explanatory power regarding the set of physiological responses, as suggested by Marston et al. (2017) [20].

Compared to the VL indexes, the TL indexes based on dimension reduction (i.e., the first dimension of PCA Dim.1, represented by the first PC, and a stacked representation of the first two PCs) come with a broader consideration of exercise and naturally better explain all the physiological responses. In practice, performing a PCA over a set of TL methods and training-related parameters is relatively accessible and could be implemented in software for coaches (e.g., athlete management systems). Using the coordinates of individuals projected on the first PC could, thus, be a more suitable way of quantifying and monitoring TL than using a single raw index (see Dim.1 in Table 3).

However, making the most of PCs through a stacked representation [72] may imply building a model on top of these PCs (see Stacked.Dim in Table 3), and hence measuring physiological or neuromuscular parameters to calibrate the model correctly. Yet, technological progress and its democratisation in resistance training support the use of affordable measurement systems in ecological conditions and would not constitute a limit in the near future.

In addition, weighting objective TL indexes by a generic neuromuscular impairment (${\mathrm{TL}}_{\mathrm{RFD}\;},\;TL_{RFD_{d}}$ and ${\mathrm{TL}}_{\mathrm{RFD}*}$) as shown in Figure 4 and Equations (3)–(5), has also substantially contributed to explaining the main physiological responses to resistance exercise when combined with other variables in PCs (see Figure 6). However, their validity remains to be further investigated, particularly under ecological conditions and with a larger number of measurements.

Among the indexes compared so far, the Borg RPE, the category ratio subjective scales (CR10) [73] and session RPE (sRPE) could stand valuable as a unique index of TL [18]. That is in line with the literature since their robustness and relevance in athlete monitoring purposes have been proven [4]. However, sRPE likely failed to explain neuromuscular responses to exercise when compared with its alternatives. It suggests that sRPE may not be the most appropriate RPE-based feature to illustrate a neuromuscular response to exercise. A possible explanation could be an interaction effect between RPE and the number of repetitions that could be blurred by the overall session duration, suggesting that physiological outcomes and RPE relationships differ between testing sessions. In addition, the pertinence of considering the time for estimating an RPE-based index (i.e., sRPE) has been questioned by authors [74].

This study has several limitations, nevertheless. First, our results apply to localised exercises performed in highly controlled conditions where the participants achieved concentric contractions of knee extensors in an open kinetic chain setting. While this experimental setting ensures a comprehensive analysis of physiological acute responses at the muscle, it might only represent a part of the overall responses underpinning resistance exercises, which could be performed in ecological conditions with polyarticular and conventional movements. In addition, measurement errors could persist and bring noise to the observed changes. Although the Biodex System 3 is widely regarded as the reference standard among isokinetic devices and appears reliable, it remains prone to measurement issues at high angular velocities [75,76]. Beyond this, even if local responses (i.e., at the muscle level) are successfully measured, systemic responses are likely to be underestimated. In addition, heterogeneous T-V profiles were observed among the participants (ranging from hyperbolic and double-hyperbolic to likely linear profiles). Despite our profile modelling methodology being recognised as valid [34], we could expect mainly quasi-linear profile shapes if modelled from multi-joint exercises [77] and considering valid measurements. Accordingly, differences in physiological responses to exercise between participants may be somewhat lowered. Also, even with three minutes of passive rest between exercises, the volume, despite being randomised, may have induced fatigue effects and impaired the T-V profiles. Yet, it does not discredit the relevance of force–velocity (or T-V) profiling for training programming. In addition, the population of interest included four females and eleven males. This discrepancy may have contributed to greater variability in the data making it more difficult to find all significant differences and correlations. Further investigations remain necessary to determine the relationships between the TL quantification methods and physiological responses underpinning resistance exercises in ecological conditions.

As a final note, the relevance of the TL indexes is essential for athlete monitoring applied to performance improvement and injury prevention [78]. In this study, we provided objective weighted TL indexes based on neuromuscular impairments following resistance exercise. Then, we compared them to former TL indexes and showed how they could be integrated into a multidimensional approach to human adaptations to RT. Using a broader set of information—through objective and subjective TL estimations, scheduling, environment, and other training-related factors—would ensure, or at least allow for a thorough understanding of individual responses to exercise for training programming and decision support. In this multidimensional perspective, providing critical insights regarding athletic performance and injuries through key performance indexes and influencing factors is essential. Therefore, dealing with different sources of information requires an appropriate modelling methodology (e.g., dimension reduction methods for high dimensionality and multicollinearity issues) to investigate relationships and causal pathways between a phenomenon and a set of explanatory features with consistency [79]. That usually implies a multidisciplinary and close collaboration between sports scientists and data scientists, mainly when the phenomenon of interest is highly complex (e.g., the injury occurrence) [80] and where its relationship with training indicators is not straightforward [78]. Linking TL estimations to athletic injuries in a unidimensional or restrictive framework may result in the identification of spurious correlations rather than the delineation of the actual causal pathways of training effects and injury occurrence [72,78]. It, thus, emphasises the importance of a multidimensional and systemic approach to understanding an athlete’s response to exercise.

## 5. Conclusions

In the present study, we measured a set of physiological responses to isolated resistance exercises to provide a more relevant and objective index of TL. Individual muscular properties were considered in the testing through individual torque–velocity profiles. Our results mainly show that at the muscle level, the current objective TL indexes suffer from a simplistic representation of exercises, whereas a more comprehensive approach better describes physiological outcomes. Accordingly, a generic equation of TL based on objective quantification methods and neuromuscular impairment contributes to a greater understanding of the physiological responses to resistance exercise. However, our prime results should be supported by further investigations involving polyarticular resistance exercises in ecological conditions.

In conclusion, a condensed representation of the various TL indexes and training-related parameters consistently reflected individual responses to exercise. In order to achieve an accurate differentiation of human responses to exercise, it is essential to consider the complex and multidimensional nature of human adaptations, as well as the concurrent objective and subjective estimates of TL.

## 6. Practical Applications

Force–velocity profiles strongly impact physiological responses to isolated lower limb resistance exercises and should constitute the basis for individual training programming.

The conventional and objective methods of training load quantification are limited to explaining physiological responses. Considering the muscle fatigue onset using a generic exponential function contributes to a more relevant expression of objective training load indexes and is supported by the observed physiological processes. It would apply to any resistance training sessions with or without biomechanical measurement systems, using the generic function $TL_{RFD}=V\;I\left(\frac{1}{e^{\alpha \;I}}\right)$ where V is the number of repetitions performed,  I denotes the relative intensity, and α the rate decay such as α=−0.071.

However, as responses to resistance exercises are heterogeneous and complex, it is not sufficient to consider training load indexes in isolation; they should be considered in conjunction with other methods and training parameters. As an alternative approach, dimension reduction methods, such as principal component analysis, are a valuable tool for compressing information into a single or a few features that can serve as a surrogate for traditional training load indexes. This observational study paves the way for further investigations into ecological conditions. However, the proposed objective methods and training load indexes are applicable in real-world settings and can contribute to a deeper understanding of the athletic response to training for monitoring purposes.

## Figures and Tables

**Table 1:** Parameter inference regarding RFD and IMP responses to exercise.

Effect	Parameter	β	Std.Error	${\mathrm{CI}}_{\mathrm{lower}}$	${\mathrm{CI}}_{\mathrm{upper}}$	DV
Population	Intercept	1142.37	2.29	929.16	1341.80	${\mathrm{RFD}}_{\mathrm{peak}}$
Population	MI	202.23	0.20	163.75	240.54	
Population	HI	224.24	0.22	201.44	287.24	
Population	$N_{\mathrm{rep}}$	−68.50	0.47	−120.16	−15.30	
Population	Gender_F	−272.17	2.72	−563.10	39.37	
Population	$N_{\mathrm{rep}}$:MI	154.90	0.21	114.48	194.96	
Population	$N_{\mathrm{rep}}$:HI	109.53	0.26	60.94	158.37	
Group	sd(ID_Intercept)	366.17	1.81	239.77	560.38	
Group	$\mathrm{sd}({\mathrm{ID}\_N}_{\mathrm{rep}})$	92.30	0.43	57.79	146.57	
Group	$\mathrm{Cor}(\mathrm{ID}\_\mathrm{Intercept}\_N_{\mathrm{rep}})$	0.08	0.00	−0.45	0.58	
Summary	PVE_Intercept	0.66		0.46	0.83	
Summary	PVE_Slope	0.05		0.01	0.11	
Summary	ICC	0.69		0.50	0.85	
Summary	$R^{2}$	0.75		0.73	0.76	
Population	Intercept	690.08	1.65	542.01	832.44	${\mathrm{RFD}}_{0–100}$
Population	MI	316.27	0.17	283.78	349.31	
Population	HI	386.82	0.21	349.47	424.49	
Population	$N_{\mathrm{rep}}$	−66.49	0.37	−108.96	−24.27	
Population	Gender_F	−243.31	2.24	−461.34	0.22	
Population	$N_{\mathrm{rep}}$:MI	121.86	0.18	88.13	155.12	
Population Group	$N_{\mathrm{rep}}$:HIsd(ID_Intercept)	72.92 250.71	0.22 1.15	31.13 168.64	114.67 280.74	
Group	$\mathrm{sd}({\mathrm{ID}\_N}_{\mathrm{rep}})$	75.20	0.34	47.60	117.77	
Group	$\mathrm{Cor}(\mathrm{ID}\_\mathrm{Intercept}\_N_{\mathrm{rep}})$	−0.34	0.00	−0.75	0.18	
Summary	PVE_Intercept	0.57		0.38	0.76	
Summary	PVE_Slope	0.06		0.02	0.13	
Summary	ICC	0.60		0.41	0.78	
Summary	$R^{2}$	0.74		0.72	0.76	
Population	Intercept	42.71	0.84	−18.92	101.92	IMP
Population	MI	107.75	0.10	88.10	127.84	
Population	HI	578.33	0.11	556.55	600.71	
Population	$N_{\mathrm{rep}}$	−2.20	0.28	−24.44	20.51	
Population	Gender_F	−2.64	0.57	−69.30	65.57	
Population	$N_{\mathrm{rep}}$:MI	1.50	0.10	−10.19	22.50	
Population	$N_{\mathrm{rep}}$:HI	14.72	0.12	−10.30	39.46	
Group	sd(ID_Intercept)	112.56	0.53	77.55	165.66	
Group	$\mathrm{sd}({\mathrm{ID}\_N}_{\mathrm{rep}})$	37.64	0.18	24.18	57.36	
Group	$\mathrm{Cor}(\mathrm{ID}\_\mathrm{Intercept}\_N_{\mathrm{rep}})$	−0.96	0.00	−1.00	−0.81	
Summary	PVE_Intercept	0.43		0.28	0.61	
Summary	PVE_Slope	0.05		0.02	0.08	
Summary	ICC	0.46		0.29	0.65	
Summary	$R^{2}$	0.83		0.82	0.83	

**Table 2:** Parameter inference regarding distributions of averaged MDF ($\bar{\mathrm{MDF}}$) and MDF rate decay (${\mathrm{MDF}}_{\alpha }$) at exercise.

Effect	Parameter	β	Std.Error	${\mathrm{CI}}_{\mathrm{lower}}$	${\mathrm{CI}}_{\mathrm{upper}}$	DV
Population	Intercept	73.64	0.05	68.95	78.37	$\bar{\mathrm{MDF}}$
Population	MI	−3.16	0.02	−5.77	−0.56	
Population	HI	−3.30	0.02	−6.01	−0.42	
Population	N	−0.45	0.00	−0.61	−0.29	
Population	MI:N	0.50	0.00	0.29	0.72	
Population	HI:N	0.42	0.00	0.15	0.72	
Group	sd(ID_Intercept)	8.56	0.03	5.76	12.83	
Group Group	sd(${\mathrm{ID}\_N}_{\mathrm{rep}}$) Cor(ID_Intercept_$N_{\mathrm{rep}})$	0.19 −0.18	0.00 0.00	0.03 −0.74	0.37 0.57	
Population	Intercept	−0.42	00.00	−1.15	0.29	${\mathrm{MDF}}_{\alpha }$
Population	MI	−0.33	0.00	−1.24	0.37	
Population	HI	−2.37	0.00	−3.08	−1.65	
Group	sd(ID_Intercept)	0.64	0.00	0.21	1.15	

**Table 3:** Summary of the variance explained by linear relationships between the different training load indexes and the main physiological responses (DV) to resistance exercise. Only estimates likely different from 0 are reported along with their 95% credible intervals (CIs) and model coefficient of determination ($R^{2}$). The highest values of $R^{2}\;$ are displayed in bold.

Method	Estimate	${\mathrm{CI}}_{\mathrm{lower}}$	${\mathrm{CI}}_{\mathrm{upper}}$	$R^{2}$	${\mathrm{CI}}_{\mathrm{lower}}$	${\mathrm{CI}}_{\mathrm{upper}}$	DV
Dens.RPE	1.85	0.58	3.13	0.32	0.06	0.55	${\mathrm{RFD}}_{0–100}$
${\mathrm{dens}\_\mathrm{RPE}}_{\mathrm{CR}10}$	4.25	0.12	8.12	0.26	0.02	0.54	${\mathrm{RFD}}_{0–100}$
Dim.1	−4.85	−7.07	−2.62	0.45	0.20	0.63	${\mathrm{RFD}}_{0–100}$
RPE	−3.60	−5.85	−1.44	0.32	0.08	0.52	${\mathrm{RFD}}_{0–100}$
${\mathrm{RPE}}_{\mathrm{CR}10}$	−4.26	−6.75	−1.78	0.35	0.11	0.53	${\mathrm{RFD}}_{0–100}$
Stacked.Dim	0.92	0.50	1.33	**0.57**	0.35	0.69	${\mathrm{RFD}}_{0–100}$
Dens.RPE	2.04	0.75	3.30	0.37	0.10	0.59	$\alpha {\mathrm{RFD}}_{\mathrm{peak}}$
${\mathrm{dens}\_\mathrm{RPE}}_{\mathrm{CR}10}$	4.24	0.40	7.88	0.26	0.03	0.53	$\alpha {\mathrm{RFD}}_{\mathrm{peak}}$
Dim.1	−5.32	−7.31	−3.34	0.58	0.34	0.73	$\alpha {\mathrm{RFD}}_{\mathrm{peak}}$
RPE	−4.04	−6.17	−1.91	0.41	0.15	0.60	$\alpha {\mathrm{RFD}}_{\mathrm{peak}}$
${\mathrm{RPE}}_{\mathrm{CR}10}$	−4.61	−6.97	−2.19	0.42	0.16	0.62	$\alpha {\mathrm{RFD}}_{\mathrm{peak}}$
${\mathrm{sRPE}}_{\mathrm{CR}10}$	−0.18	−0.35	−0.02	0.23	0.03	0.44	$\alpha {\mathrm{RFD}}_{\mathrm{peak}}$
Stacked.Dim	0.95	0.62	1.28	**0.68**	0.52	0.76	$\alpha {\mathrm{RFD}}_{\mathrm{peak}}$
Dens.RPE	0.11	0.05	0.17	0.28	0.08	0.46	$\alpha \bar{MDF}$
${\mathrm{dens}\_\mathrm{RPE}}_{\mathrm{CR}10}$	0.24	0.05	0.43	0.18	0.03	0.36	$\alpha \bar{MDF}$
Dim.1	−0.34	−0.41	−0.26	0.69	0.51	0.79	$\alpha \bar{MDF}$
RPE	−0.28	−0.39	−0.16	0.49	0.23	0.67	$\alpha \bar{MDF}$
${\mathrm{RPE}}_{\mathrm{CR}10}$	−0.32	−0.45	−0.19	0.51	0.25	0.68	$\alpha \bar{MDF}$
Stacked.Dim	1.02	0.82	1.22	**0.77**	0.69	0.81	$\alpha \bar{MDF}$
${\mathrm{dens}\_\mathrm{RPE}}_{\mathrm{CR}10}$	0.30	0.11	0.49	0.58	0.35	0.71	$\left[lact_{p}\right]$
Dim.1	−0.14	−0.26	−0.01	0.56	0.31	0.70	$\left[lact_{p}\right]$
Stacked.Dim	0.77	0.33	1.20	**0.75**	0.67	0.80	$\left[lact_{p}\right]$
${\mathrm{dens}\_\mathrm{mdl}}_{RFD}$	−0.48	−0.82	−0.14	0.28	0.05	0.53	EE_tot

## Data Availability

The datasets analysed for this study can be made available upon demand.

## Supplementary Materials

The following supporting information can be downloaded at: https://www.mdpi.com/article/10.3390/sports13010013/s1, Figure S1. Distribution of regression slopes for changes in (a) RFD_peak_ and (b) RFD_0–100_ across repetitions of knee extensions. Conditions 1, 2, and 3 refer to LI, MI, and HI testing sessions. Figure S2. Distribution of regression slopes for changes in median frequencies from power spectrum (MDF_α_) across repetitions of isokinetic knee extensions. Conditions 1, 2, and 3 refer to LI, MI, and HI testing sessions. Figure S3. Distribution of (a) rate decay of Δ[Hb_diff_] and (b) rate decay of TSI at exercise. Conditions 1, 2, and 3 refer to LI, MI, and HI testing sessions. Table S1. Configuration of the three-knee extension testing sessions. Table S2. Posterior estimates regarding then normalised averaged torque produced at exercise. β denotes standardised regression coefficients. The model summary includes proportion of variance explained (PVE), intraclass correlation coefficient (ICC) and R^2^. Table S3. Posterior estimates regarding the distributions of summated EMG signals at exercise. The model summary includes proportion of variance explained (PVE), intraclass correlation coefficient (ICC) and R^2^. Table S4. Posterior estimate regarding changes in blood lactate concentrations ([lact_p_]) in response to exercise. The model summary includes proportion of variance explained (PVE), intraclass correlation coefficient (ICC) and R^2^. Table S5. Posterior estimate regarding changes in blood lactate concentrations ([cort_p_]) in response to exercise. The model summary includes proportion of variance explained (PVE), intraclass correlation coefficient (ICC) and R^2^.

## Glossary

$\left[cort_{p}\right]$	plasma cortisol concentration;
$\left[lact_{b}\right]$	blood lactate concentration;
$\Delta \left[{\mathrm{Hb}}_{\mathrm{diff}}\right]$	muscle oxygenation index;
${\mathrm{CO}}_{2}$	carbon dioxide;
EE	net energy expenditure;
EMG	electromyography;
IMP	mechanical impulse;
LI	low intensity;
LMM	linear mixed models;
MI	moderate intensity;
$m\dot{V}O_{2}$	muscle oxygen consumption;
HI	high intensity;
HR	heart rate;
MDF	median frequency;
MVC	maximum voluntary contraction;
NIRS	near-infrared spectroscopy;
$O_{2}$	oxygen;
PC	principal component;
PCA	principal component analysis;
RFD	rate of force development;
${\mathrm{RFD}}_{0–100}$	rate of force development over the first 100 ms of exercise;
${\mathrm{RFD}}_{\mathrm{peak}}$	peak rate of force development reached over a repetition;
RFem	rectus femoris;
RM	repetition maximum;
RMS	root mean square;
RPE	rate of perceived exertion;
RT	resistance training;
SRS	spatially resolved spectroscopy;
STFT	short-term Fourier transform;
TL	training load;
TSI	tissue saturation index;
TUT	time under tension;
T-V	torque–velocity;
VMed	vastus medialis;
VL	volume-load;
VLat	vastus lateralis;
$\dot{V}O_{2}$	net oxygen uptake.
