# Supplementary material for: A Muscle Physiology-Based Framework for Quantifying Training Load in Resistance Exercises
Source: Sports (Basel). 2025 Jan 9;13(1):13. doi: 10.3390/sports13010013 (PMC11768794; doi:10.3390/sports13010013)
Supplement: Supplementary file 1 [file sports-13-00013-s001.zip › sports-3289818-supplementary.pdf]

Title: A Muscle Physiology-Based Framework for Quantifying Training Load in Resistance Exercises†

Running title: Physiology-based framework for training load

Frank Imbach[1,2,3], Stéphane Perrey[2*], Thomas Brioche[3], and Robin Candau[3]

[1] Seenovate, Montpellier, 34000, France

[2] EuroMov Digital Health in Motion, Univ Montpellier, IMT Mines Alès, Montpellier, France

[3] DMeM, Univ Montpellier, INRAE, Montpellier, France

*Corresponding author: Stephane Perrey – stephane.perrey@umontpellier.fr

† This is a part of PhD thesis of Frank Imbach in University of Montpellier, Montpellier, FR

Supplementary Materials

Tables

Table S1. Configuration of the three-knee extension testing sessions

Session	Sets	Repetitions	Intensity (% MVC)	Passive recovery (s)
1	1	24	58 %	N/A
2	2	9	77 %	180
3	5	3	93 %	240

Table S2. Posterior estimates regarding then normalised averaged torque produced at exercise. β denotes the posterior mean of the standardised regression coefficients. The model summary includes proportion of variance explained (PVE), intraclass correlation coefficient (ICC) and R^2.

Effect	Parameter	β	Std.error	$\mathrm{CI}_{\mathrm{lower}}$	$\mathrm{CI}_{\mathrm{upper}}$
Population-level	Intercept	24.33	0.11	14.60	34.16
Population-level	MI	15.05	0.01	14.16	15.93
Population-level	HI	22.75	0.01	-2.91	23.73
Population-level	$\mathrm{N}_{\mathrm{rep}}$	-1.85	0.01	-2.91	-0.74
Population-level	Gender_M	0.64	0.11	-9.90	10.91
Population-level	$\mathrm{N}_{\mathrm{rep}}$:MI	2.27	0.00	1.41	3.15
Population-level	$\mathrm{N}_{\mathrm{rep}}$:HI	3.08	0.01	2.02	4.13
Group-level	sd(ID_Intercept)	11.47	0.06	7.93	16.99
Group-level	sd(ID_$\mathrm{N}_{\mathrm{rep}}$)	1.86	0.01	1.18	2.90
Group-level	Cor(ID_Intercept_$\mathrm{N}_{\mathrm{rep}}$)	-0.62	0.00	-0.89	-0.17
Summary	PVE_Intercept	0.82		0.70	0.91
Summary	PVE_slope	0.02		0.01	0.05

Summary	ICC	0.84		0.73	0.93
Summary	R^2	0.89		0.88	0.89

Table S3. Posterior estimates regarding the distributions of summated EMG signals at exercise. β denotes the posterior mean of the standardised regression coefficients. The model summary includes proportion of variance explained (PVE), intraclass correlation coefficient (ICC) and R^2.

Effect	Parameter	β	Std.error	$\mathrm{CI}_{\mathrm{lower}}$	$\mathrm{CI}_{\mathrm{upper}}$
Population-level	Intercept	432.75	0.30	401.52	462.03
Population-level	MI	-35.93	0.05	-45.60	-26.25
Population-level	HI	-92.67	0.06	-103.27	-82.17
Population-level	$\mathrm{N}_{\mathrm{rep}}$	12.56	0.11	2.45	23.01
Population-level	Gender_M	-20.41	0.21	-47.86	8.45
Population-level	$\mathrm{N}_{\mathrm{rep}}$:MI	-7.47	0.05	-17.11	2.17
Population-level	$\mathrm{N}_{\mathrm{rep}}$:HI	-12.27	0.06	-24.02	-0.49
Group-level	sd(ID_Intercept)	41.20	0.17	28.12	62.05
Group-level	sd(ID_$\mathrm{N}_{\mathrm{rep}}$)	16.78	0.07	10.41	26.42
Group-level	Cor(ID_Intercept_$\mathrm{N}_{\mathrm{rep}}$)	0.92	0.00	0.68	1.00
Summary	PVE_Intercept	0.34		0.20	0.53
Summary	PVE_slope	0.06		0.02	0.11

Summary	ICC	0.36		0.21	0.57
Summary	R^2	0.54		0.50	0.57

Table S4. Posterior estimate regarding changes in blood lactate concentrations ($[lact_p]$) in response to exercise. β denotes the posterior mean of the standardised regression coefficients. The model summary includes proportion of variance explained (PVE), intraclass correlation coefficient (ICC) and R^2.

Effect	Parameter	β	Std.error	CI_{lower}	CI_{upper}
Population-level	Intercept	2.98	0.01	1.61	4.29
Population-level	MI	-1.83	0.01	-3.05	-0.59
Population-level	HI	-8.30	0.06	-13.07	-3.13
Population-level	Velocity	-0.61	0.01	-1.48	0.35
Population-level	Sexe	0.31	0.01	-0.88	1.44
Population-level	MI:velocity	-1.53	0.01	-2.99	-0.04
Population-level	HI:velocity	-6.68	0.06	-11.79	-1.16
Group-level	sd(ID_Intercept)	0.81	0.01	0.26	1.46
Group-level	sd(ID_Velocity)	0.59	0.01	0.05	1.30
Group-level	Cor(ID_Intercept_Velocity)	-0.46	0.01	-0.98	0.61
Summary	PVE_Intercept	0.32		0.04	0.66
Summary	PVE_slope	0.20		0.00	0.57

Summary	ICC	0.41		0.05	0.75
Summary	R^2	0.69		0.49	0.83

Table S5. Posterior estimate regarding changes in blood lactate concentrations ($[cort_p]$) in response to exercise. β denotes the posterior mean of the standardised regression coefficients. The model summary includes proportion of variance explained (PVE), intraclass correlation coefficient (ICC) and R^2.

Effect	Parameter	β	Std.error	$\mathrm{CI}_{\mathrm{lower}}$	$\mathrm{CI}_{\mathrm{upper}}$
Population-level	Intercept	0.40	0.07	-9.49	10.16
Population-level	MI	-0.87	0.08	-11.23	9.86
Population-level	HI	-1.78	0.10	-16.71	-13.37
Population-level	Velocity	-0.90	0.07	-9.94	8.04
Population-level	Gender	0.04	0.03	-3.41	3.49
Population-level	MI:velocity	1.14	0.09	-13.60	15.83
Population-level	HI:velocity	1.12	0.11	-15.42	17.65
Group-level	sd(ID_Intercept)	1.66	0.02	0.16	3.42
Group-level	sd(ID_Velocity)	4.86	0.02	2.85	7.67
Group-level	Cor(ID_Intercept_Velocity)	0.49	0.01	-0.47	7.67
Summary	PVE_Intercept	0.09		0.00	0.28
Summary	PVE_slope	0.62		0.31	0.84
Summary	ICC	0.24		0.00	0.64

Summary	R^2	0.65		0.37	0.82

Figures

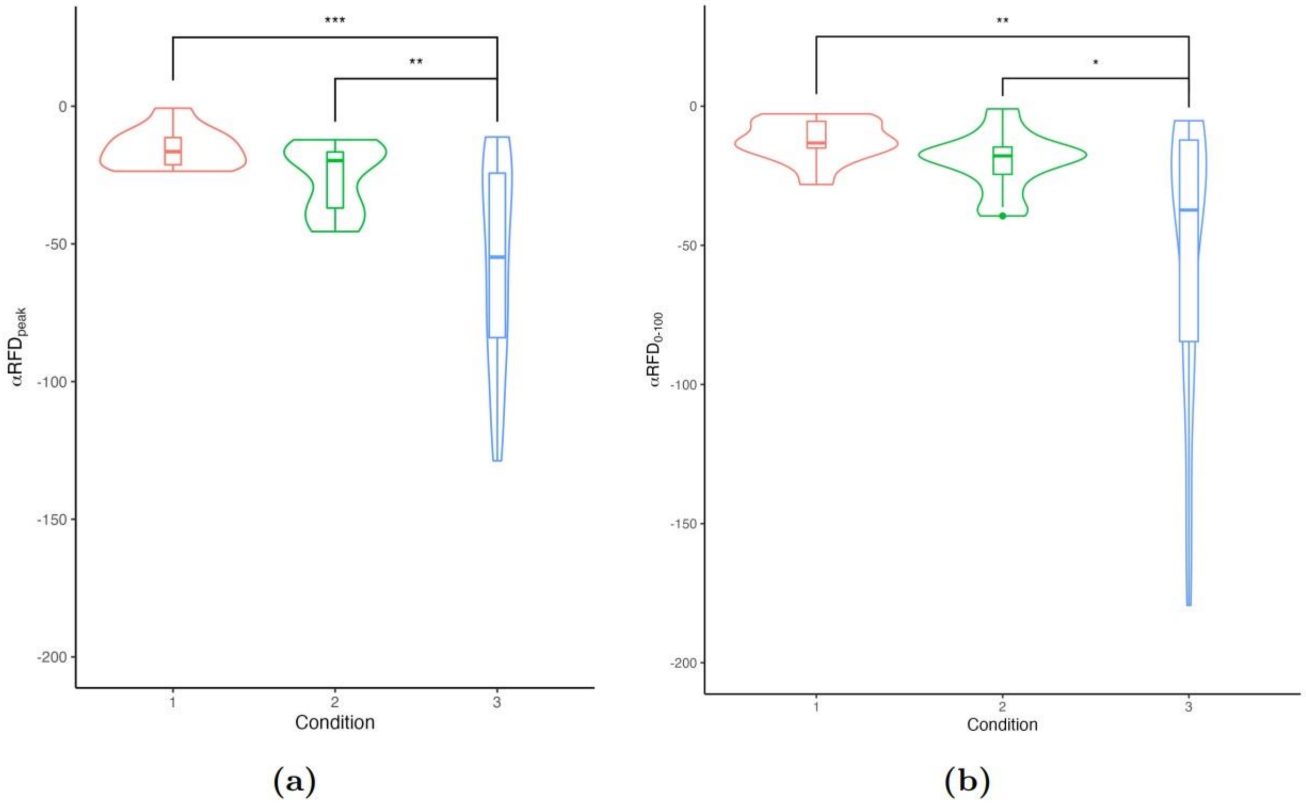

Figure S1. Distribution of regression slopes for changes in (a) RFD_{peak} and (b) RFD_{0-100} across repetitions of knee extensions. Conditions 1, 2, and 3 refer to LI, MI, and HI testing sessions.

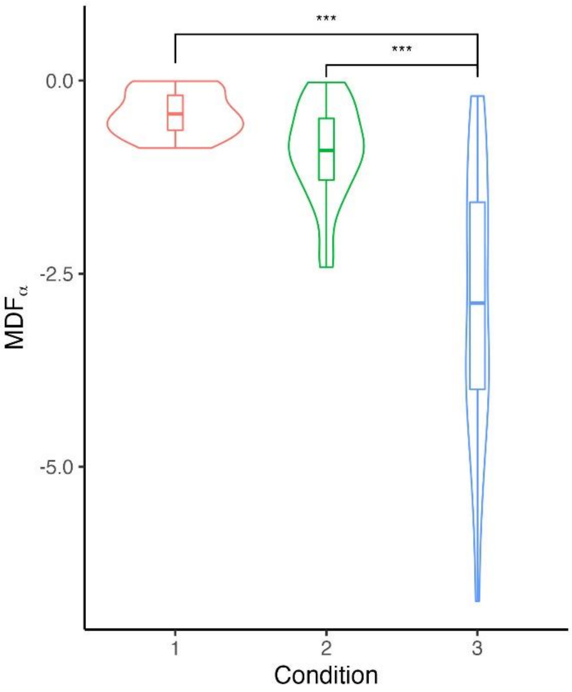

Figure S2. Distribution of regression slopes for changes in median frequencies from power spectrum (MDF_{α}) across repetitions of isokinetic knee extensions. Conditions 1, 2, and 3 refer to LI, MI, and HI testing sessions.

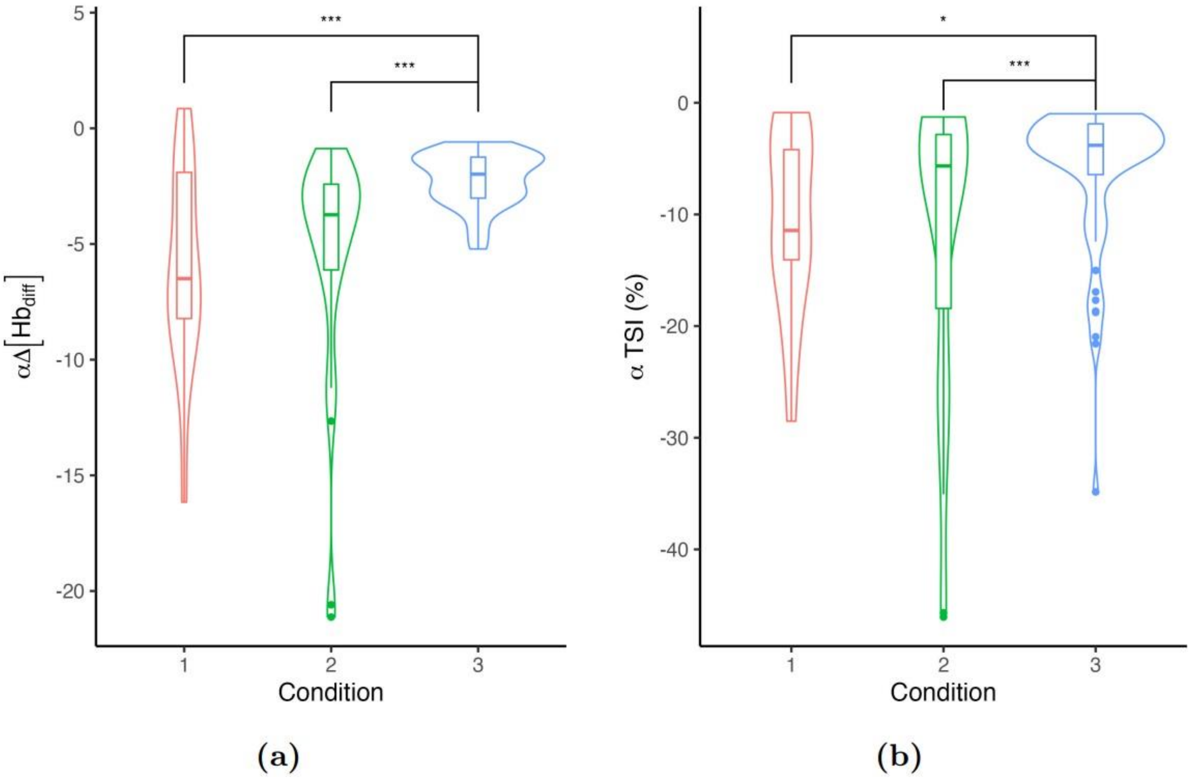

Figure S3. Distribution of (a) rate decay of $\Delta[\mathrm{Hb_{diff}}]$ and (b) rate decay of TSI at exercise. Conditions 1, 2, and 3 refer to LI, MI, and HI testing sessions.
